# Implementation of Option B and a fixed-dose combination antiretroviral regimen for prevention of mother-to-child transmission of HIV in South Africa: A model of uptake and adherence to care

**DOI:** 10.1371/journal.pone.0201955

**Published:** 2018-08-30

**Authors:** Mhairi Maskew, Lise Jamieson, Given Mohomi, Lawrence Long, Constance Mongwenyana, Cynthia Nyoni, Dorah Mokaba, Matthew P. Fox, Ian Sanne, Sydney Rosen

**Affiliations:** 1 Health Economics and Epidemiology Research Office, Department of Internal Medicine, School of Clinical Medicine, Faculty of Health Sciences, University of the Witwatersrand, Johannesburg, South Africa; 2 Department of Global Health, Boston University School of Public Health, Boston, Massachusetts, United States of America; 3 City of Johannesburg, Johannesburg, South Africa; 4 Department of Epidemiology, Boston University School of Public Health, Boston, Boston University School of Public Health, Boston, Massachusetts; Universita degli Studi di Roma Tor Vergata, ITALY

## Abstract

**Introduction:**

Initiating and retaining pregnant women on antiretroviral therapy (ART) to prevent mother-to-child HIV transmission (PMTCT) remains a major challenge facing African HIV programs, particularly during the critical final months prior to delivery. In 2013, South Africa implemented its “Option B” PMTCT regimen (three-drug ART throughout pregnancy and breastfeeding, regardless of maternal CD4 count) and introduced once-daily fixed-dose combinations and lifelong ART. Currently, the uptake of Option B and its possible impact on adherence to PMTCT during the critical final months of pregnancy is unclear.

**Materials and methods:**

We prospectively collected visit data from a cohort of adult, HIV-infected, pregnant women between July 2013-August 2014 to estimate three models of adherence to PMTCT during the final 16 weeks immediately preceding delivery. Adherence was defined according to possession of antiretroviral drugs, which was inferred from clinic visit records under varying assumptions in each model. We describe uptake of the PMTCT regimen, gestational age at initiation, and model possible scenarios of adherence through delivery after the implementation of Option B.

**Results:**

Among 138 women enrolled (median (IQR) age 28 years (24–32), median CD4 count 378 cells/mm^3^), median (IQR) gestational age at initiation was 22 weeks (16–26). Estimates of adherence during the final 16 weeks of pregnancy prior to delivery ranged from 75% (52–89%) under the best case scenario assumptions to 52% (30–75%) under the worst case scenario assumptions. Estimates of the proportion of women who would achieve 80% adherence to PMTCT were <50% across all models.

**Conclusions:**

Despite the switch to Option B and once-daily dosing, South African women continue to initiate PMTCT late in pregnancy, and estimations of regimen adherence, as modelled using PMTCT visit attendance data, is poor, with <50% of women reaching 80% adherence during final months of pregnancy across all models. Further guideline changes and interventions are needed to achieve vertical transmission goals.

**Trial registration:**

ClinicalTrials.gov NCT01710397

South African National Clinical Trials Register DOH-27-0213-4177

## Introduction

Initiating and retaining HIV-infected pregnant women on a regimen of antiretroviral medications for prevention of mother-to-child transmission of HIV (PMTCT) has long been one of the major challenges facing African HIV programs. Although transmission of HIV to infants has fallen sharply in many countries [1,2], continued investment and significant resources are still required to improve PMTCT delivery [3]. In South Africa, as in other countries in the region, late presentation for antenatal care, low uptake of medications, and poor adherence to the prescribed regimen persist in antenatal PMTCT programs [4–7].

In April 2013, South Africa replaced its earlier and more complicated PMTCT regimen, known as “Option A,” with the World Health Organization-recommended “Option B” [8]. Under Option B, all pregnant women were offered three-drug ART for the duration of pregnancy and breastfeeding, regardless of the mother’s baseline CD4 cell count. Simultaneously, a once daily fixed dose combination tablet was introduced in PMTCT programs to replace the two- or three- tablet, twice-daily formulation previously used for the standard first-line regimen of tenofovir, 3TC or FTC, and efavirenz [9]. Taken together, these changes meant that a woman receiving a positive HIV test result at her first antenatal visit could walk out the door the same day with a simple ARV regimen that would remain constant until she ceased breastfeeding. Under the guidelines in effect prior to 2013, South Africa’s success in preventing vertical transmission was impressive but incomplete [2,10]. The goal of both of the changes described above was to simplify and therefore improve adherence to ARVs and further reduce risk of MTCT by creating the opportunity for sustained viral suppression for the duration of antenatal care attended during pregnancy, ultimately moving the country closer to elimination of vertical transmission.

Quantifying the impact such changes might have on adherence to PMTCT and subsequent viral suppression during pregnancy, however, remains challenging. Data systems in resource constrained environments often do not capture medication dispensing records and adherence is often estimated through observational proxy data sources such as antenatal visit attendance data. This method may be limited by several challenges: 1) Recorded attendance at an antenatal visit may not mean that medication was dispensed, as linked dispensing records are rarely kept; 2) pregnant women frequently deliver at a different facility from the one at which they received antenatal care, leaving the ANC clinic without a record of the actual date of delivery; and 3) women who stop attending care at the original facility are designated as lost from care, despite evidence that many of these women have self-transferred to other facilities [11]. When this happens, clinic visits attended during the later period of PMTCT prior to delivery are not observed.

These limitations may result in over- or under-estimation of adherence to PMTCT, particularly during the critical period immediately prior to delivery. To help understand adherence to PMTCT after South Africa’s switch to Option B and once-daily, single-tablet dosing, we collected observational data describing gestational age at ARV initiation, and visit attendance from initiation through delivery for a cohort of HIV-infected pregnant women at a public sector primary health clinic after the change in guidelines to Option B. These data were then used to parameterize three hypothetical models of adherence to PMTCT in order to quantify a possible range of adherence given uncertainty around medication dispensing practices at observed antenatal visits. We also estimate potential predictors of adherence under these models.

## Materials and methods

### Study site, population, and data

The data utilized in this study were collected at a public-sector primary health clinic (PHC) in Johannesburg, South Africa. It is located in an informal settlement on the edge of the city with a large population of immigrants from neighboring countries and patients from other South African provinces. It provides a full range of primary healthcare services, including antenatal care (ANC), maternal and child health, HIV testing and counseling, ART initiation, and ART management. Under South African guidelines], women are advised to make at least four antenatal care visits prior to delivery, starting at 14 weeks’ gestation. PMTCT guidelines increased the required number of visits, however, as clinics standard practice was to provide only one month of ARV medication at a time during the study period. The clinic does not have a delivery ward; referral for delivery was to either a nearby hospital or one of three maternal obstetric units (MOUs) located 4, 5.5, and 7 kilometers from the clinic, respectively. At least one antenatal clinic visit was needed for a woman to obtain an antenatal care card, which is required to assure access to a hospital or MOU for delivery.

We sequentially enrolled adult (≥18 years old), HIV-infected, pregnant women at the earliest clinic visit for a positive HIV test, provision of a blood sample for a CD4 count, or first ANC visit between July 4, 2013 and August 14, 2014. Women who were already on ART for their own health or who indicated that they did not intend to seek the remainder of their antenatal care at the study clinic were excluded. After informed consent, the women were administered a questionnaire eliciting information about demographic and socioeconomic characteristics; previous pregnancies, health-seeking behavior and exposure to PMTCT; normal activities and employment, and the costs the patient incurred per clinic visit. Study patients were then followed by passive medical record review, using the site’s and delivery facility’s routine patient records, until delivery. These records included antenatal and labour ward registries as well as an electronic medical record system, TherapyEdge-HIV^™^. Fields collected included dates of antenatal clinic visits, gravidity and parity, gestational age at first visit and predicted delivery date, date of HIV test, ARV regimen prescribed, and baseline CD4 count. Not all data for all study subjects could be found in the routine records maintained by the study site, nor could confirmation of all deliveries be found at the referral facilities. Viral load test results were not available for the majority of study participants, as most had spent fewer than 6 months on ART at the time of delivery.

### Primary outcome

The primary defined outcome for this study was proportion of patients achieving 80% adherence to PMTCT in the 16 weeks immediately preceding date of delivery. For this analysis, we assumed 16 weeks to be a reasonable minimum duration of ART needed to achieve a high rate of viral suppression during pregnancy based on published South African data [12,13] and approximately 80% adherence to PMTCT to be the minimum required for successful viral suppression [14,15]. We focused the analysis on the final four months (16 weeks) before delivery on the assumption that viral suppression during this later period is more important for reducing the risk of MTCT [13] than earlier in the pregnancy. Adherence to PMTCT was estimated using clinic visit attendance under three different models with varying assumptions.

### Model assumptions

Date of delivery was unknown for some of the women in the study. We instead assumed that for those without a known delivery date, delivery took place at either 1) 36 weeks’ gestation; 2) 38 weeks’ gestation; or 3) 40 weeks’ gestation. Based on these assumptions, delivery dates were imputed accordingly based on the gestational age at which a woman presented at her first antenatal visit.

Using the data described above, we then estimated three models with varying assumptions about medication dispensed at each observed antenatal clinic visit (Table 1). Because we cannot observe actual adherence, we used medication possession to proxy adherence, assuming that if a 28-day supply of medication was dispensed, then the patient could be considered fully adherent for the next 28 days [16]. In Model A (100% dispensing), we assumed that all women with a documented clinic visit received 28 days’ worth of medication at each visit (i.e., that the stipulated clinic practice was adhered to perfectly) except the last documented visit, as explained below. For Model B (90% dispensing) we assume that 90% of women who attended a visit received 28 days’ worth of medication while the remaining 10% attended a visit but were not dispensed medication. To model this, 90% of women were randomly allocated 28 days’ medication while the remaining 10% were allocated 0 days’ medication at each visit except the last documented visit, and women who were allocated 0 days’ medication at a given visit were not permitted to be randomized to the 0 days’ medication allocation at the next visit, so that that no woman attended two consecutive visits without receiving medication at one of the visits at a minimum. Model C (80% dispensing) followed a similar structure to Model B: at each visit except the last documented visit, 80% of women were randomly allocated 28 days’ medication while the remaining 20% were allocated 0 days’ medication. Again, we assumed that no women attended two consecutive visits without receiving medication during at least one of the visits. For any visit that occurred < 28 days prior to a subsequent visit (in other words, an unscheduled visit prior to a routine scheduled visit), we allocated the women sufficient pills for the gap between the visits and then continued the model allocation at the subsequent (scheduled) visit.

**Table 1:** Model assumptions.

Model	Primary Assumption for pill dispensing	Levels of estimated delivery date**
Model A– 100% pill dispensing	All women who attend a clinic visit receive 28 days’ worth of pills at each documented visit except the last documented visit*	36 weeks
		38 weeks
		40 weeks
Model B– 90% pill dispensing	Assume 90% of women who attended a visit got 28 days’ worth of pills while the remaining 10% attended a visit but were not dispensed pills except for the last documented visit*.	36 weeks
		38 weeks
		40 weeks
Model C– 80% pill dispensing	Assume 80% of women who attended a visit got 28 days’ worth of pills while the remaining 20% attended a visit but were not dispensed pills except for the last documented visit*.	36 weeks
		38 weeks
		40 weeks

* All models—Last visit pill dispensing: Women with observed clinic visits up to delivery date or estimated delivery date are allocated 28 days’ worth of pills at the last visit according to the primary assumption for each model (100%, 90% or 80% dispensing). Women with > one month unobserved time prior to delivery date or estimated delivery date—assume 40% of these women remain in care during unobserved time ((they get sufficient pills up to known or estimated delivery date) while 60% get the allocated 28 days’ worth of pills dispensed at their last visit according to the model allocation (100%, 90% or 80% dispensing).

** Assumption applied to n = 34 women with unknown delivery date

We handled the last observed visit differently. For women with observed clinic visits within one month of their delivery date or estimated delivery date, medication dispensing continued according to the primary assumption for each model (Model A—100% women got 28 days’ allocation, Model B—90% women got 28 days’ allocation, or Model C—80% women got 28 days’ allocation). For women with more than one month of unobserved time prior to their delivery date (i.e., those who were considered lost from care more than one month prior to their known or estimated delivery date), however, several possibilities exist for medication adherence during the unobserved time. First, a woman may truly be lost from care and not taking any medication during the entire period. Second, the woman may have transferred to another facility, such as the maternity unit where she intended to deliver, and continued treatment at the new facility. Finally, the woman may have been in possession of some medication from her previous visits, due to less than full adherence earlier in the pregnancy, and continued to take the leftover medications until that supply was depleted.

A previous report from South Africa estimated that almost 40% of women who appeared lost from care at the original antenatal care facility were in fact receiving on-going care at another facility where they ultimately delivered]. To account for this, in all models we adjusted dispensing at the last observed visit among women with more than one-month unobserved time prior to delivery date or estimated delivery date (i.e. those apparently lost to care). We randomly selected 40% of these women and assumed that they remained in care during the entire period of unobserved time prior to delivery, while the remaining 60% got only the medications dispensed at their last visit according to the model allocation (Model A—100% women got 28 days’ pill dispensing, Model B—90% women got 28 days’ pill dispensing, or Model C—80% women got 28 days’ pill dispensing).

### Data analysis

We initially summarized characteristics at study enrolment with descriptive statistics. Next, for each of the three models’ set of assumptions, we estimated the proportion of women considered adherent to PMTCT during the final 16 weeks immediately preceding delivery using medians and interquartile ranges (IQR). We then categorized adherence for each woman in the last 16 weeks of pregnancy (weeks 24 to 40) as >80% or not and described the proportion of women achieving 80% adherence during this period for each of the levels in the three models. Finally, we fitted a log-binomial regression model to estimate the risk ratios (and 95% confidence intervals) of potential baseline predictors of achieving 80% adherence to PMTCT during the final 16 weeks of pregnancy.

#### Trial registration

ClinicalTrials.gov NCT01710397

South African National Clinical Trials Register DOH-27-0213-4177

This study was nested within the original RapIT randomized controlled trial which was registered on ClinicalTrials.gov (Registration number) NCT01710397 on September 7, 2012 and the South African National Clinical Trials Register DOH-27-0213-4177. The first participant in the RapIT study was enrolled on May 8th, 2013.

All study participants provided written informed consent and the consent procedure as well as all other study procedures received ethics approval from the institutional review boards of Boston University and the University of the Witwatersrand.

## Results

### Enrolment and patient characteristics

**Fig 1:**
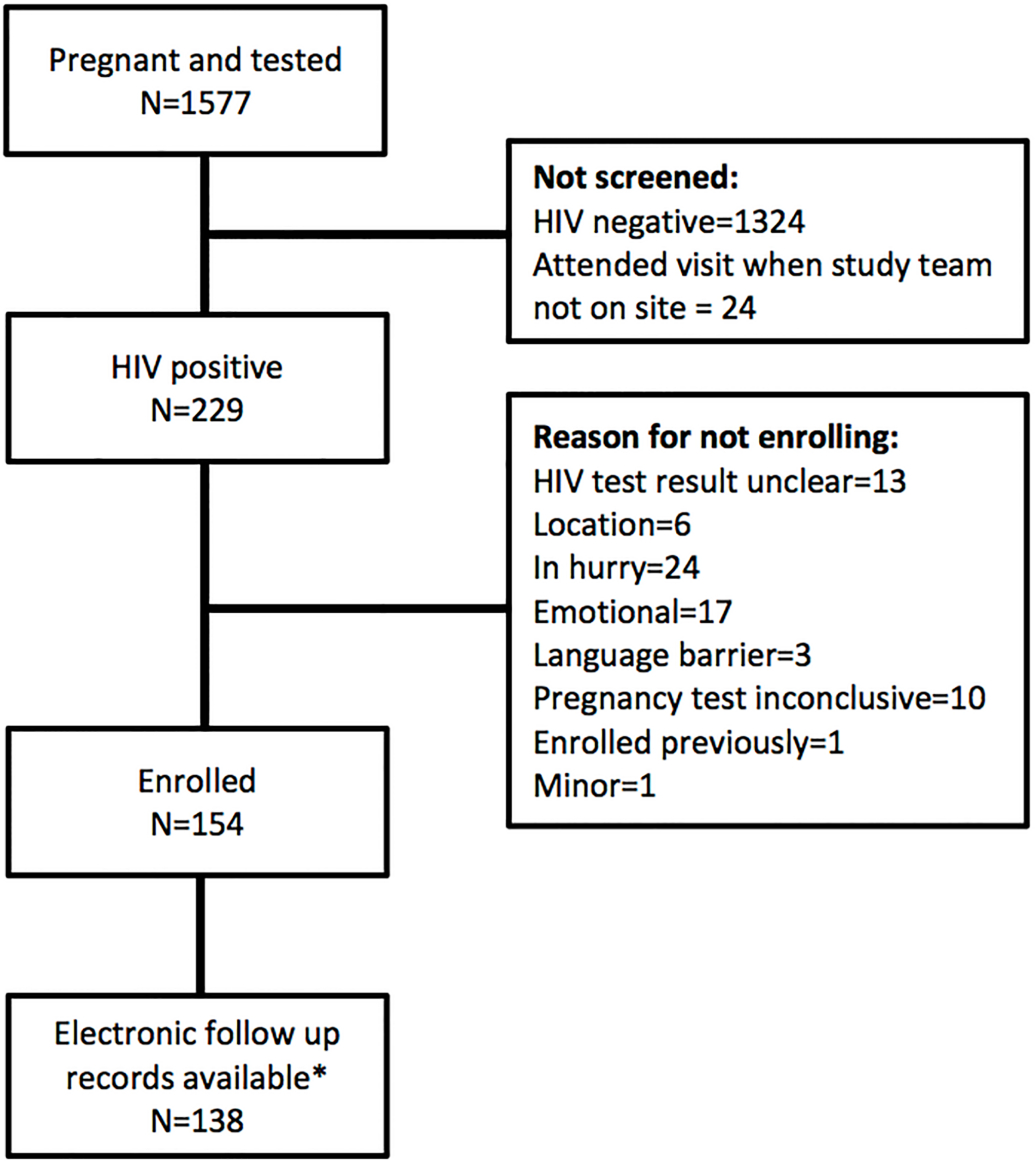
Study enrolment flow chart.

During the period July 2013 to August 2014, a total of 1577 pregnant women were tested for HIV at the study site or, if already known to be positive, were not yet on antiretroviral medications and had a CD4 count and were potentially eligible for study enrolment. Of these, 24 attended their booking visit on days that the study team was not on site. Two hundred twenty-nine women of the remaining 1553 (15%) were confirmed HIV positive and screened for study enrollment; 154/229 (61%) met inclusion criteria and were enrolled. Of the 75 screened but not enrolled, 37 were ineligible for the study, 24 refused to participate, 10 had an inconclusive pregnancy test, 3 were unable to be enrolled due to language barriers, and 1 was already enrolled in the study. After excluding 16 patients whose follow-up medical records were incomplete, we included 138 patients in the final data sample (Fig 1).

Patient characteristics are summarized in Table 2. The women enrolled were a median of 28 years of age (IQR 24–32) with a median gravidity of 2 (2–3) and parity of 1 (1–2). The median presenting CD4 count was 378 cells/mm^3^ and nearly half presented with CD4>350 cells/mm^3^ (45%). Nearly all (>90%) reported having previously tested for HIV. Most either lived in informal housing (44%) or in a single room in shared accommodation (46%). Fewer than 20% reported being formally employed and most (77%) walked to the clinic. Only 2 women (1%) reported having a child who was also HIV positive. Delivery dates were available for 104 (75%) of women enrolled.

**Table 2:** Baseline characteristics of study sample (n = 138).

Characteristic	Unit or response	Value
Pregnancy and PMTCT* characteristics		
Estimated gestational age at first recorded PMTCT drug collection (weeks)	Median (IQR*)	22 (16–26)
Gravidity	Median (IQR)	2 (2–3)
Parity	Median (IQR)	1 (1–2)
CD4 count at first recorded PMTCT* drug collection (cells/mm^3^)	Median (IQR)	378 (252–523)
CD4 count category at first recorded PMTCT drug collection (cells/mm^3^)	Missing	24 (17%)
	<200	17 (12%)
	201–350	35 (26%)
	>350	62 (45%)
Source of data on delivery date	Clinic or delivery facility records	104 (75%)
	Imputed from gestational age at booking	34 (25%)
Personal and household characteristics		
Age	Median (IQR)	28 (24–32)
Housing type	Independent house	14 (10%)
	Single room in shared accommodation	64 (46%)
	Informal dwelling/shack	60 (44%)
Duration living there	< 1 year	44 (32%)
	1–5 years	45 (33%)
	>5 years	49 (35%)
HIV diagnosis	Ever tested before today	125 (91%)
	Positive results before today	44 (32%)
	Diagnosed first time today	94 (69%)
Other HIV-positive individual in household	N, %	22 (16%)
Other individual on ART in household	N, %	21 (32%)
Marital status	Single	1 (1%)
	Long-term partner	107 (77%)
	Married	29 (21%)
	Widowed	1 (1%)
Employment status	Formal job	26 (19%)
	Informal work	16 (11%)
	Unemployed, seeking work	80 (58%)
	Unemployed, not seeking work	16 (12%)
Getting to the clinic	Group (minibus) taxi	30 (22%)
	Walking	106 (77%)
	Private car	2 (1%)
	Incurred any transport cost	32 (23%)
	If any transport cost, median (IQR) cost each way (Rand)	8 (8–8)

* PMTCT—prevention of mother to child transmission; IQR—interquartile range

### Access to PMTCT care

The median gestational age at PMTCT medication initiation was 22 weeks (IQR 16–26 weeks). Just 13% of women had made an initial visit—whether for an HIV test, CD4 count, or antenatal care—by 14 weeks’ gestation, which is the PMTCT starting date recommended by guidelines. More than a third (38%) attended their first antenatal visit and initiated PMTCT medications <16 weeks before their estimated delivery date. Overall, the median number of antenatal clinic visits between first visit and delivery was 4 (IQR 2–5); most of these visits occurred during the later stages of pregnancy with a median of 3 visits (IQR 2–4) made during the last 16 weeks immediately preceding delivery.

After applying model assumptions for dispensing at each visit, the observed visit schedule resulted in a median of 14 weeks (IQR 10–18 weeks), 13 weeks (IQR 8–17 weeks) and 12 weeks (IQR 6–16 weeks) of estimated PMTCT drug possession between initiation and delivery for Model A, Model B, and Model C, respectively.

### Adherence to PMTCT during 16 weeks prior to delivery

**Fig 2:**
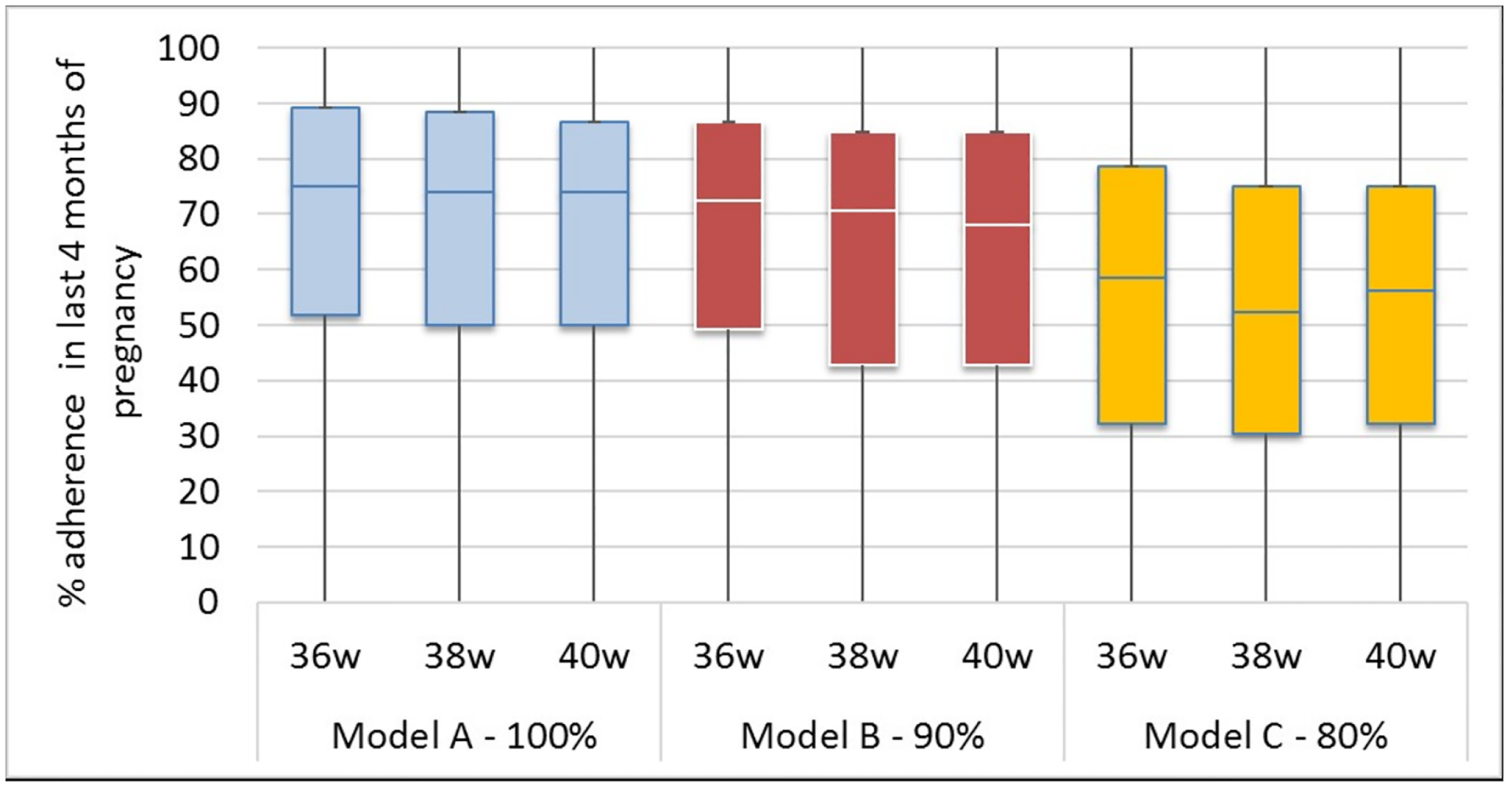
Estimated adherence to PMTCT care during four months immediately preceding delivery stratified by model.

Under Model A, during the last four months before delivery estimates of adherence were 75% (52–89%), 74% (50–88%) and 74% (50–87%) for the 36-week, 38- week and 40-week delivery intervals, respectively (Fig 2). Under the Model B, the corresponding estimates of adherence dropped to 72% (49–87%), 71% (43–85%) and 68% (43–85%) for the 36-week, 38- week and 40-week delivery intervals. Adherence fell even further under Model C to <60% for all levels: 59% (32–79%), 52% (30–75%) and 56% (32–75%) for the 36-week, 38- week and 40-week delivery intervals, respectively.

**Fig 3:**
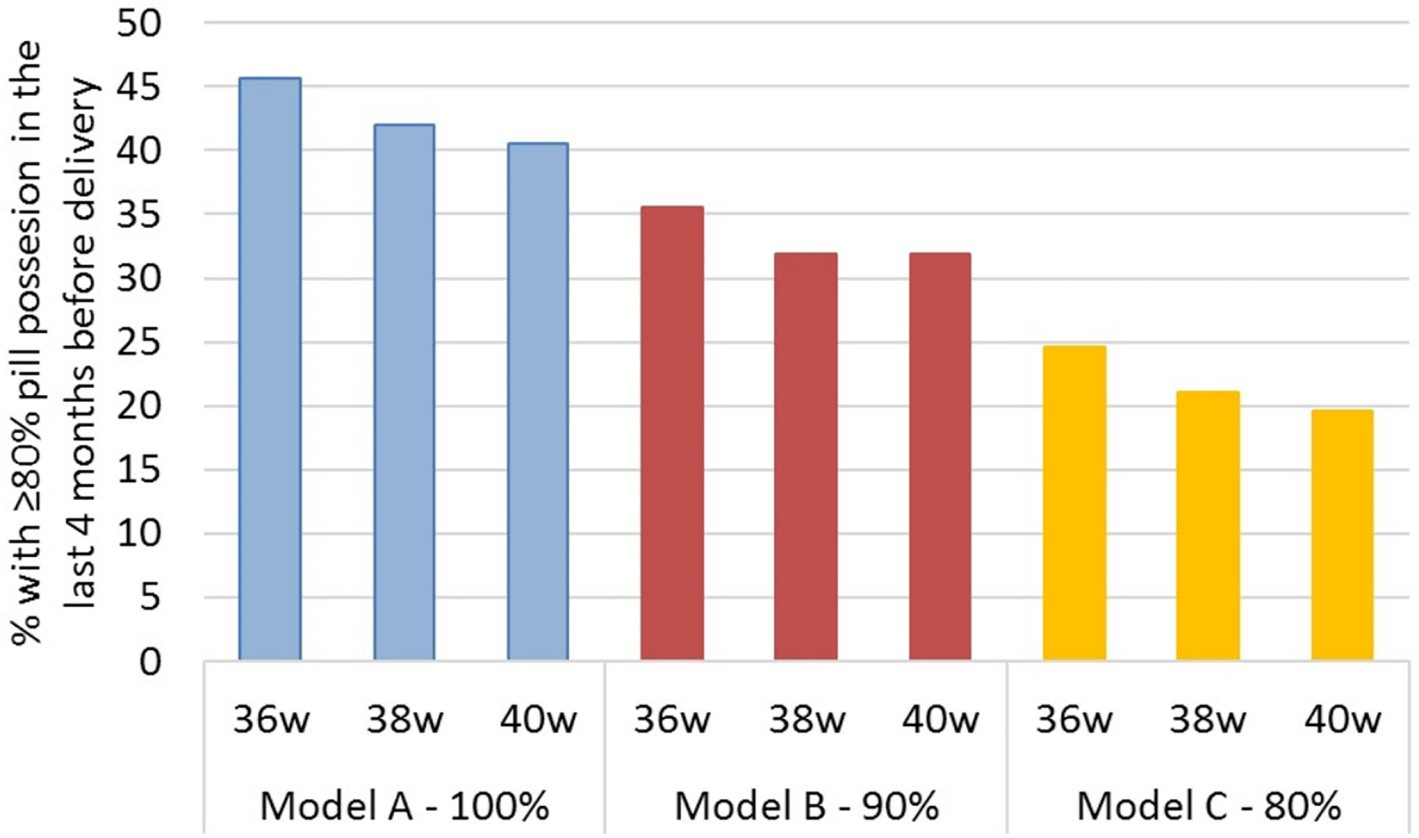
Estimates of proportion of women who may achieve 80% adherence during four months immediately preceding delivery stratified by model.

Based on the primary data collected, the modeled proportion of women achieving 80% adherence during the final 16 weeks prior to delivery was low for all models (Fig 3). Under Model A, the estimated proportion of women who achieved 80% adherence was 46%, 42%, and 41% for the 36-week, 38- week and 40-week delivery intervals, respectively; this dropped to 36%, 32%, and 32% for the same intervals under Model B and to 25%, 21%, and 20% for the 36-week, 38- week and 40-week delivery intervals, respectively, under Model C.

### Predictor analysis

We also estimated predictors of 80% adherence to PMTCT during the final four months of pregnancy prior to delivery under each of the three models. Though our estimates lacked precision, results from all three models suggested that younger (aged <28 years), employed, or married women or those who had more than one child previously would more frequently achieve 80% adherence to PMTCT during the four months prior to delivery when compared to older, unemployed, or single women without a long-term partner or those who had either no or only one previous child (S1 Tables 1–3 and S1 Fig). In addition, those women who had tested HIV positive prior to booking at antenatal clinic and those who attended their first antenatal clinic visit at or before 22 weeks’ gestation also may be more likely to achieve 80% adherence, except under model C conditions which suggested that those booking after 22 weeks’ may be more likely to achieve the 80% adherence goal. CD4 count at entry to antenatal care did not appear to affect adherence under Model A conditions, but, though not consistent for all levels of assumption, those with CD4 >350 cells/m^3^ may more frequently achieve 80% adherence to PMTCT under Model B or C conditions.

## Discussion

Encouraging more HIV-infected pregnant women to initiate antiretroviral medications earlier in pregnancy and remain adherent throughout the pregnancy is essential to achieving global goals for the elimination of mother-to-child transmission. The guideline change introduced by South Africa in 2013 aimed to do just this, by offering a simpler standard ARV regimen that could be initiated on the day of the woman’s first clinic visit, even before the formal start of antenatal care. In this description of adherence to PMTCT medications after the guideline change, we found that gaps remain in the critical parameters that influence risks of vertical transmission—gestational age at regimen initiation, adherence to the regimen for the duration of pregnancy, and adherence during the crucial final four months of pregnancy. Most women in our cohort initiated ARVs late, at a median (IQR) gestational age of 22 (16–26) weeks, nearly identical to the median of 24 (19–28) weeks reported for an earlier cohort enrolled at the same study site under the previous (Option A) guidelines [17].

Based on antenatal clinic visit data, the modelled estimates of adherence to PMTCT during the critical final 16 weeks preceding delivery were well below desired levels. Even under the best-case scenario assumptions where every woman received medication at every attended visit (Model A), fewer than half of women were estimated to achieve 80% adherence during this critical period. Under worst-case assumptions, as few as one fifth did so. All estimates suggested that there is still a good deal of room for improvement in adherence.

We suggest three reasons that adherence to PMTCT continued to be poor, despite the changes made in 2013. First, neither of the policy changes introduced under Option B+ was intended to reduce gestational age at ARV initiation. The changes were imposed upon an environment in which women typically make their first antenatal visit well into the second trimester of pregnancy [4]. Second, the new guidelines did not affect the absolute number of clinic visits that pregnant women must make, nor improve their access to points of care. While taking a single, fixed-dose combination tablet once a day is certainly easier than taking multiple tablets twice a day, it does not address the barrier imposed by monthly clinic visits for medication refills, which are often not timed to be coincident with regular antenatal care visits. Finally, the guideline changes did not address known behavioral or facility barriers to PMTCT initiation or adherence, ranging from stigma to poor quality of care [17–21]. Though our models estimating predictors of adherence lacked precision, the results suggested that women entering antenatal care newly diagnosed with HIV may struggle to achieve ideal adherence levels compared to those women whose status was known prior to the pregnancy. Investigations into the benefit of increased support for women entering both HIV and antenatal care programs may be warranted.

Our analysis had a number of limitations. Our estimates were based on assumptions around PMTCT medication possession data as observed in clinic visit records and not actual dispensing data, and while we believe our assumptions reflect clinic practice during the study period (one months’ medications dispensed per visit), it is possible that these did not correlate precisely. If two or more months’ medication were actually dispensed, our model would under-estimate adherence. We also note that the PMTCT regimen adherence we observed was disturbingly low and appears inconsistent with national reports of sharply lower vertical transmission of HIV [22]. We speculate that this in part reflects the phenomenon of “silent transfers”—women who choose to relocate during pregnancy to a distant geographic area and deliver near family or other social support. These women re-start PMTCT at a new clinic without informing the initiating clinic, and thus appear as lost to follow up in the initiating clinic’s records. This problem is one of the major challenges confronting researchers aiming to estimate ART retention and adherence globally. We attempted to account for this by adjusting the assumptions on the last visit for those not observed until delivery based on data from a recent study that tracked women considered to be lost from PMTCT care which reported that overall loss to care fell by a third if transfers to other clinics, both nearby and in other provinces were counted [11]. But to the extent that women may resume PMTCT care and deliver somewhere else (beyond the 40% we accounted for), our models underestimate PMTCT adherence. Finally, the data used in the models represented women attending one antenatal site in Johannesburg and, as such, may not be representative of the experience of all women across South Africa.

## Conclusions

Implementation of Option B and once-daily dosing of antiretrovirals does not appear to have increased early uptake of PMTCT among pregnant women in South Africa. Women in this study continued to present late in pregnancy for PMTCT, and adherence to the regimen, across three varying models of medication possession, appeared to be poor, with fewer than half of women reaching 80% adherence during the final months of pregnancy. Although the adoption of “test and treat” guidelines for HIV in 2016 may continue to increase the proportion of women already on ART at conception, there will continue to be many women whose diagnosis of HIV occurs at an antenatal visit. Further research into barriers to accessing early ANC and PMTCT among pregnant women as well as interventions to support both earlier initiation of PMTCT medications and continued adherence to them until (and after) delivery are needed to achieve vertical transmission goals.

## Supporting information

S1 Fig Predictors of adherence to PMTCT stratified by model. (TIF)

S1 Tables Predictors of achieving 80% adherence to PMTCT under Model A, B and C assumptions. (PDF)
