# Supplementary figures and images for: Implementation of Option B and a fixed-dose combination antiretroviral regimen for prevention of mother-to-child transmission of HIV in South Africa: A model of uptake and adherence to care
Source: PLoS One. 2018 Aug 30;13(8):e0201955. doi: 10.1371/journal.pone.0201955 (PMC6116946; doi:10.1371/journal.pone.0201955)

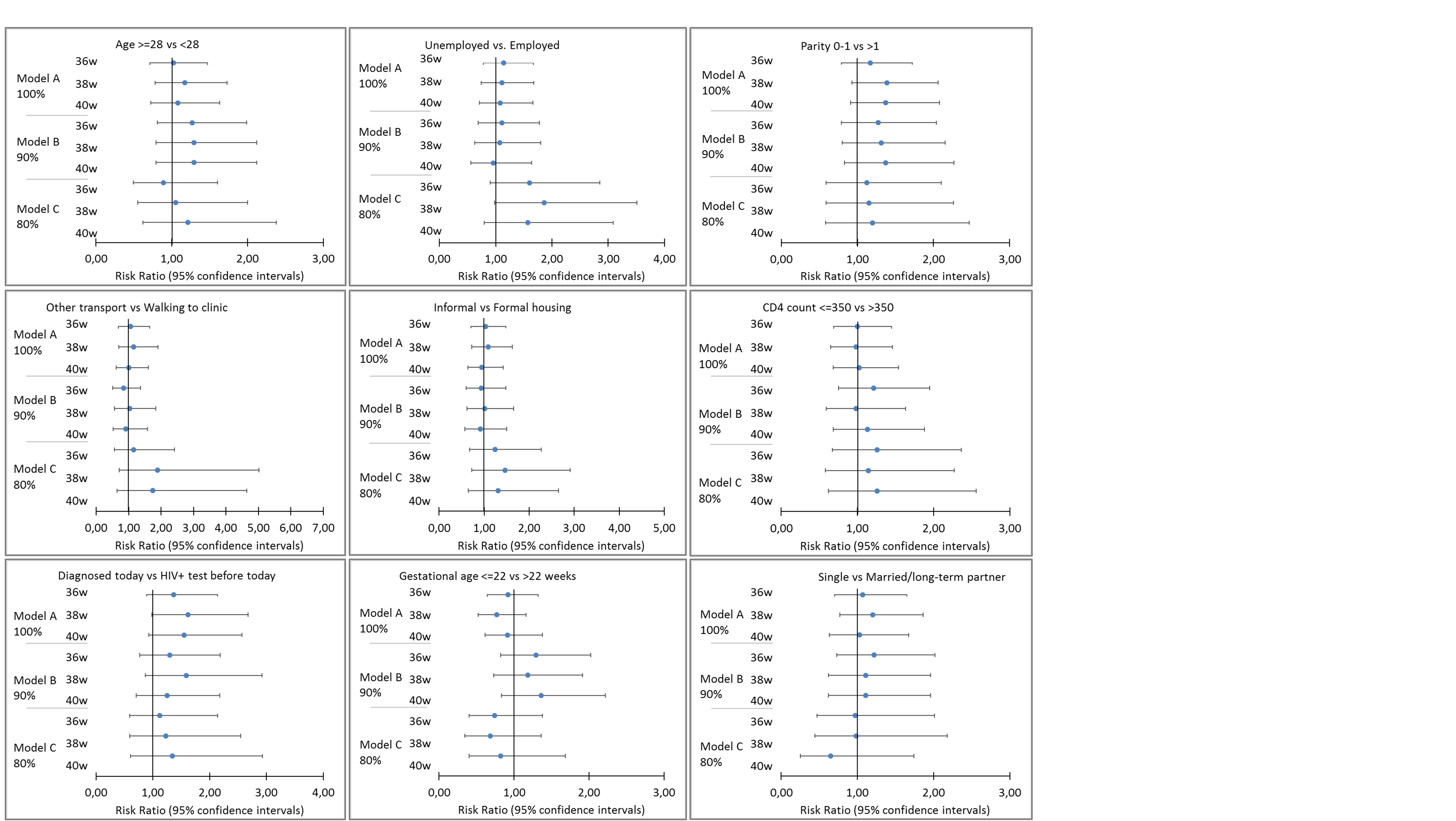

Supplement: S1 Fig — (TIF) [file pone.0201955.s001.tif]
