# Supplementary material for: Implementation of Option B and a fixed-dose combination antiretroviral regimen for prevention of mother-to-child transmission of HIV in South Africa: A model of uptake and adherence to care
Source: PLoS One. 2018 Aug 30;13(8):e0201955. doi: 10.1371/journal.pone.0201955 (PMC6116946; doi:10.1371/journal.pone.0201955)
Supplement: S1 Tables — (PDF) [file pone.0201955.s002.pdf]

Supplementary Table 1: Predictors of achieving 80% adherence to PMTCT under Model A assumptions (100% pill dispensing)

Gestational age at delivery	Variable	Category	N	n (%) with 80% adherence	Unadjusted Risk Ratio (95% CI)	Adjusted Risk Ratio (95% CI)
36 weeks	Age (in years)	>=28	72	45.8% (33)	1.00 (reference)	1.00 (reference)
		<28	64	46.9% (30)	1.02 (0.71-1.47)	0.92 (0.60-1.41)
	Gestational age at booking	<=22	69	50.7% (35)	1.00 (reference)	1.00 (reference)
		>22	56	46.4% (26)	0.92 (0.64-1.32)	0.84 (0.55-1.26)
	Parity	0-1	77	42.9% (33)	1.00 (reference)	
		>1	46	50.0% (23)	1.17 (0.79-1.72)	
	Married	No	109	45.0% (49)	1.00 (reference)	
		Yes	29	48.3% (14)	1.07 (0.70-1.65)	
	Employed	No	96	43.8% (42)	1.00 (reference)	1.00 (reference)
		Yes	42	50.0% (21)	1.14 (0.78-1.67)	1.22 (0.77-1.94)
	CD4 count at booking	<=350	52	50.0% (26)	1.00 (reference)	1.00 (reference)
		>350	62	50.0% (31)	1.00 (0.69-1.45)	0.97 (0.61-1.53)
	Housing type	Informal	60	45.0% (27)	1.00 (reference)	1.00 (reference)
		Formal	78	46.2% (36)	1.03 (0.71-1.48)	0.85 (0.53-1.35)
	Walking to clinic	No	32	43.8% (14)	1.00 (reference)	1.00 (reference)
		Yes	106	46.2% (49)	1.06 (0.68-1.65)	0.86 (0.55-1.35)
	Had a prior HIV+ test	Yes	44	36.4% (16)	1.00 (reference)	1.00 (reference)
		No	94	50.0% (47)	1.37 (0.89-2.14)	1.18 (0.71-1.94)
38 weeks	Age (in years)	>=28	72	38.9% (28)	1.00 (reference)	1.00 (reference)
		<28	64	45.3% (29)	1.17 (0.78-1.73)	1.20 (0.79-1.82)
	Gestational age at booking	<=22	69	50.7% (35)	1.00 (reference)	1.00 (reference)
		>22	56	39.3% (22)	0.77 (0.52-1.16)	0.72 (0.47-1.10)
	Parity	0-1	77	37.7% (29)	1.00 (reference)	
		>1	46	52.2% (24)	1.39 (0.93-2.06)	
	Married	No	109	40.4% (44)	1.00 (reference)	
		Yes	29	48.3% (14)	1.20 (0.77-1.86)	
	Employed	No	96	40.6% (39)	1.00 (reference)	1.00 (reference)
		Yes	42	45.2% (19)	1.11 (0.74-1.68)	1.06 (0.69-1.63)
	CD4 count at booking	<=350	52	46.2% (24)	1.00 (reference)	1.00 (reference)
		>350	62	45.2% (28)	0.98 (0.65-1.46)	0.98 (0.64-1.51)
	Housing type	Informal	60	40.0% (24)	1.00 (reference)	1.00 (reference)
		Formal	78	43.6% (34)	1.09 (0.73-1.63)	1.11 (0.72-1.72)
	Walking to clinic	No	32	37.5% (12)	1.00 (reference)	1.00 (reference)
		Yes	106	43.4% (46)	1.16 (0.70-1.90)	1.08 (0.68-1.72)
	Had a prior HIV+ test	Yes	44	29.5% (13)	1.00 (reference)	1.00 (reference)
		No	94	47.9% (45)	1.62 (0.98-2.68)	1.57 (0.93-2.66)
40 weeks	Age (in years)	>=28	72	38.9% (28)	1.00 (reference)	1.00 (reference)
		<28	64	42.2% (27)	1.08 (0.72-1.63)	1.05 (0.64-1.72)
	Gestational age at booking	<=22	69	44.9% (31)	1.00 (reference)	1.00 (reference)
		>22	56	41.1% (23)	0.91 (0.61-1.38)	0.87 (0.56-1.36)
	Parity	0-1	77	36.4% (28)	1.00 (reference)	

		>1	46	50.0% (23)	1.37 (0.91-2.08)	
	Married	No	109	40.4% (44)	1.00 (reference)	
		Yes	29	41.4% (12)	1.03 (0.63-1.67)	
	Employed	No	96	39.6% (38)	1.00 (reference)	1.00 (reference)
		Yes	42	42.9% (18)	1.08 (0.71-1.66)	0.95 (0.56-1.60)
	CD4 count at booking visit	<=350	52	44.2% (23)	1.00 (reference)	1.00 (reference)
		>350	62	45.2% (28)	1.02 (0.68-1.54)	1.03 (0.65-1.62)
	Housing type	Informal	60	41.7% (25)	1.00 (reference)	1.00 (reference)
		Formal	78	39.7% (31)	0.95 (0.64-1.43)	1.09 (0.63-1.89)
	Walking to clinic	No	32	40.6% (13)	1.00 (reference)	1.00 (reference)
		Yes	106	40.6% (43)	1.00 (0.62-1.61)	0.95 (0.58-1.56)
	Had a prior HIV+ test	Yes	44	29.5% (13)	1.00 (reference)	1.00 (reference)
		No	94	45.7% (43)	1.55 (0.93-2.57)	1.57 (0.86-2.85)

Supplementary Table 2: Predictors of achieving 80% adherence to PMTCT under Model B assumptions (90% pill dispensing)

Gestational age at delivery	Variable	Category	N	n (%) with 80% adherence	Unadjusted Risk Ratio (95% CI)	Adjusted Risk Ratio (95% CI)
36 weeks	Age (in years)	>=28	72	31.9% (23)	1.00 (reference)	1.00 (reference)
		<28	64	40.6% (26)	1.27 (0.81-1.99)	1.08 (0.66-1.79)
	Gestational age at booking	<=22	69	33.3% (23)	1.00 (reference)	1.00 (reference)
		>22	56	42.9% (24)	1.29 (0.82-2.02)	1.21 (0.74-1.98)
	Parity	0-1	77	32.5% (25)	1.00 (reference)	
		>1	46	41.3% (19)	1.27 (0.79-2.04)	
	Married	No	109	33.9% (37)	1.00 (reference)	
		Yes	29	41.4% (12)	1.22 (0.73-2.02)	
	Employed	No	96	34.4% (33)	1.00 (reference)	1.00 (reference)
		Yes	42	38.1% (16)	1.11 (0.69-1.78)	1.20 (0.72-1.99)
	CD4 count at booking	<=350	52	34.6% (18)	1.00 (reference)	1.00 (reference)
		>350	62	41.9% (26)	1.21 (0.75-1.95)	1.06 (0.62-1.81)
	Housing type	Informal	60	36.7% (22)	1.00 (reference)	1.00 (reference)
		Formal	78	34.6% (27)	0.94 (0.60-1.48)	0.89 (0.53-1.49)
	Walking to clinic	No	32	40.6% (13)	1.00 (reference)	1.00 (reference)
		Yes	106	34.0% (36)	0.84 (0.51-1.37)	0.60 (0.35-1.02)
	Had a prior HIV+ test	Yes	44	29.5% (13)	1.00 (reference)	1.00 (reference)
		No	94	38.3% (36)	1.30 (0.77-2.19)	1.03 (0.59-1.81)
38 weeks	Age (in years)	>=28	72	27.8% (20)	1.00 (reference)	1.00 (reference)
		<28	64	35.9% (23)	1.29 (0.79-2.12)	1.53 (0.82-2.84)
	Gestational age at booking	<=22	69	31.9% (22)	1.00 (reference)	1.00 (reference)
		>22	56	37.5% (21)	1.18 (0.73-1.91)	1.38 (0.74-2.59)
	Parity	0-1	77	29.9% (23)	1.00 (reference)	
		>1	46	39.1% (18)	1.31 (0.80-2.15)	
	Married	No	109	31.2% (34)	1.00 (reference)	
		Yes	29	34.5% (10)	1.11 (0.62-1.96)	
	Employed	No	96	31.3% (30)	1.00 (reference)	1.00 (reference)
		Yes	42	33.3% (14)	1.07 (0.63-1.80)	1.05 (0.54-2.02)
	CD4 count at booking	<=350	52	34.6% (18)	1.00 (reference)	1.00 (reference)
		>350	62	33.9% (21)	0.98 (0.59-1.63)	0.96 (0.54-1.70)
	Housing type	Informal	60	31.7% (19)	1.00 (reference)	1.00 (reference)
		Formal	78	32.1% (25)	1.01 (0.62-1.66)	1.34 (0.71-2.54)
	Walking to clinic	No	32	31.3% (10)	1.00 (reference)	1.00 (reference)
		Yes	106	32.1% (34)	1.03 (0.57-1.84)	0.77 (0.41-1.48)
	Had a prior HIV+ test	Yes	44	22.7% (10)	1.00 (reference)	1.00 (reference)
		No	94	36.2% (34)	1.59 (0.87-2.92)	1.51 (0.73-3.11)
40 weeks	Age (in years)	>=28	72	27.8% (20)	1.00 (reference)	1.00 (reference)
		<28	64	35.9% (23)	1.29 (0.79-2.12)	1.32 (0.71-2.43)
	Gestational age at booking	<=22	69	29.0% (20)	1.00 (reference)	1.00 (reference)
		>22	56	39.3% (22)	1.36 (0.83-2.22)	1.44 (0.78-2.65)
	Parity	0-1	77	28.6% (22)	1.00 (reference)	

		>1	46	39.1% (18)	1.37 (0.83-2.27)	
	Married	No	109	31.2% (34)	1.00 (reference)	
		Yes	29	34.5% (10)	1.11 (0.62-1.96)	
	Employed	No	96	32.3% (31)	1.00 (reference)	1.00 (reference)
		Yes	42	31.0% (13)	0.96 (0.56-1.64)	0.97 (0.51-1.87)
	CD4 count at booking visit	<=350	52	32.7% (17)	1.00 (reference)	1.00 (reference)
		>350	62	37.1% (23)	1.13 (0.68-1.88)	0.99 (0.56-1.77)
	Housing type	Informal	60	33.3% (20)	1.00 (reference)	1.00 (reference)
		Formal	78	30.8% (24)	0.92 (0.57-1.50)	1.06 (0.55-2.02)
	Walking to clinic	No	32	34.4% (11)	1.00 (reference)	1.00 (reference)
		Yes	106	31.1% (33)	0.91 (0.52-1.58)	0.72 (0.39-1.34)
	Had a prior HIV+ test	Yes	44	27.3% (12)	1.00 (reference)	1.00 (reference)
		No	94	34.0% (32)	1.25 (0.71-2.18)	1.13 (0.57-2.27)

Supplementary Table 3: Predictors of achieving 80% adherence to PMTCT under Model C assumptions (80% pill dispensing)

Gestational age at delivery	Variable	Category	N	n (%) with 80% adherence	Unadjusted Risk Ratio (95% CI)	Adjusted Risk Ratio (95% CI)
36 weeks	Age (in years)	>=28	72	26.4% (19)	1.00 (reference)	1.00 (reference)
		<28	64	23.4% (15)	0.89 (0.49-1.60)	0.63 (0.31-1.29)
	Gestational age at booking	<=22	69	29.0% (20)	1.00 (reference)	1.00 (reference)
		>22	56	21.4% (12)	0.74 (0.40-1.38)	0.52 (0.25-1.06)
	Parity	0-1	77	23.4% (18)	1.00 (reference)	
		>1	46	26.1% (12)	1.12 (0.59-2.10)	
	Married	No	109	24.8% (27)	1.00 (reference)	
		Yes	29	24.1% (7)	0.97 (0.47-2.01)	
	Employed	No	96	20.8% (20)	1.00 (reference)	1.00 (reference)
		Yes	42	33.3% (14)	1.60 (0.90-2.85)	1.47 (0.76-2.86)
	CD4 count at booking	<=350	52	23.1% (12)	1.00 (reference)	1.00 (reference)
		>350	62	29.0% (18)	1.26 (0.67-2.36)	1.45 (0.66-3.16)
	Housing type	Informal	60	21.7% (13)	1.00 (reference)	1.00 (reference)
		Formal	78	26.9% (21)	1.24 (0.68-2.27)	1.26 (0.59-2.71)
	Walking to clinic	No	32	21.9% (7)	1.00 (reference)	1.00 (reference)
		Yes	106	25.5% (27)	1.16 (0.56-2.42)	1.08 (0.48-2.44)
	Had a prior HIV+ test	Yes	44	22.7% (10)	1.00 (reference)	1.00 (reference)
		No	94	25.5% (24)	1.12 (0.59-2.14)	0.85 (0.41-1.78)
38 weeks	Age (in years)	>=28	72	20.8% (15)	1.00 (reference)	1.00 (reference)
		<28	64	21.9% (14)	1.05 (0.55-2.00)	1.22 (0.59-2.51)
	Gestational age at booking	<=22	69	26.1% (18)	1.00 (reference)	1.00 (reference)
		>22	56	17.9% (10)	0.68 (0.34-1.36)	0.43 (0.20-0.94)
	Parity	0-1	77	20.8% (16)	1.00 (reference)	
		>1	46	23.9% (11)	1.15 (0.59-2.26)	
	Married	No	109	21.1% (23)	1.00 (reference)	
		Yes	29	20.7% (6)	0.98 (0.44-2.18)	
	Employed	No	96	16.7% (16)	1.00 (reference)	1.00 (reference)
		Yes	42	31.0% (13)	1.86 (0.98-3.51)	1.48 (0.71-3.10)
	CD4 count at booking	<=350	52	21.2% (11)	1.00 (reference)	1.00 (reference)
		>350	62	24.2% (15)	1.14 (0.58-2.27)	1.37 (0.65-2.88)
	Housing type	Informal	60	16.7% (10)	1.00 (reference)	1.00 (reference)
		Formal	78	24.4% (19)	1.46 (0.73-2.91)	2.01 (0.90-4.47)
	Walking to clinic	No	32	12.5% (4)	1.00 (reference)	1.00 (reference)
		Yes	106	23.6% (25)	1.89 (0.71-5.02)	1.98 (0.77-5.08)
	Had a prior HIV+ test	Yes	44	18.2% (8)	1.00 (reference)	1.00 (reference)
		No	94	22.3% (21)	1.23 (0.59-2.55)	1.07 (0.47-2.40)
40 weeks	Age (in years)	>=28	72	18.1% (13)	1.00 (reference)	1.00 (reference)
		<28	64	21.9% (14)	1.21 (0.62-2.38)	1.08 (0.50-2.31)
	Gestational age at booking	<=22	69	21.7% (15)	1.00 (reference)	1.00 (reference)
		>22	56	17.9% (10)	0.82 (0.40-1.68)	0.62 (0.28-1.37)
	Parity	0-1	77	18.2% (14)	1.00 (reference)	

		>1	46	21.7% (10)	1.20 (0.58-2.47)	
	Married	No	109	21.1% (23)	1.00 (reference)	
		Yes	29	13.8% (4)	0.65 (0.25-1.74)	
	Employed	No	96	16.7% (16)	1.00 (reference)	1.00 (reference)
		Yes	42	26.2% (11)	1.57 (0.80-3.09)	1.18 (0.54-2.62)
	CD4 count at booking visit	<=350	52	19.2% (10)	1.00 (reference)	1.00 (reference)
		>350	62	24.2% (15)	1.26 (0.62-2.56)	1.18 (0.53-2.60)
	Housing type	Informal	60	16.7% (10)	1.00 (reference)	1.00 (reference)
		Formal	78	21.8% (17)	1.31 (0.65-2.65)	1.43 (0.63-3.25)
	Walking to clinic	No	32	12.5% (4)	1.00 (reference)	1.00 (reference)
		Yes	106	21.7% (23)	1.74 (0.65-4.65)	2.35 (0.75-7.34)
	Had a prior HIV+ test	Yes	44	15.9% (7)	1.00 (reference)	1.00 (reference)
		No	94	21.3% (20)	1.34 (0.61-2.93)	1.22 (0.52-2.86)
